# The effect of labor and delivery mode on electrocortical and brainstem autonomic function during neonatal transition

**DOI:** 10.1038/s41598-019-47306-1

**Published:** 2019-07-30

**Authors:** Sarah B. Mulkey, Srinivas Kota, Rathinaswamy B. Govindan, Tareq Al-Shargabi, Christopher B. Swisher, Augustine Eze, Laura Hitchings, Stephanie Russo, Nicole Herrera, Robert McCarter, G. Larry Maxwell, Robin Baker, Adre J. du Plessis

**Affiliations:** 1 Children’s National Health System, Washington, DC United States; 2 Inova Women’s and Children’s Hospital, Falls Church, VA United States; 3 Fairfax Neonatal Associates, Fairfax, VA United States; 4 Departments of Pediatrics and Neurology, The George Washington University School of Medicine and Health Sciences, Washington, DC United States

**Keywords:** Development of the nervous system, Paediatric research, Neurological disorders

## Abstract

Delivery of the newborn occurs either vaginally or via caesarean section. It is not known whether the mode of delivery and exposure to labor affects early autonomic nervous system (ANS) function, as measured by heart rate variability (HRV), or cortical electroencephalogram (EEG) activity. The objective of the study was to determine if autonomic function in newborns differs by mode of delivery. Simultaneous recording of EEG and electrocardiogram were collected in low-risk term newborns at <72 hours of age to measure HRV, the asymmetry index, and EEG power. Newborns were compared by delivery type: vaginal delivery (VD), cesarean section (CS) after labor (L-CS), or elective CS (E-CS). Quantile Regression controlled for gestational age, postnatal age, and percent active states. One hundred and eighteen newborns were studied at 25.2 (11.4) hours of age. Sixty-two (52.5%) were born by VD, 22 by L-CS (18.6%), and 34 by E-CS (28.8%). HRV metrics didn’t differ by delivery mode. Asymmetry index was higher in L-CS compared to VD and E-CS (P = 0.03). On EEG, L-CS newborns showed lower relative gamma power compared to VD and E-CS (P = 0.005). The study found that overall ANS tone is not altered by mode of delivery in low-risk term newborns.

## Introduction

As the low-risk fetus nears delivery at term gestational age, the autonomic nervous system (ANS) and its cerebral connections adapt to support the infant during transition to the extra-uterine environment. At delivery, sympathetic outflow from the brainstem and from higher brain centers surges^1^ to support successful transition of the fetus to the newborn^2^. The stimulus for these changes begins during labor. Indeed, cortisol levels steadily rise toward term and then increase dramatically during labor^3,4^. With elective cesarean section without labor, however, cortisol levels may be lower than needed for optimal transition^3^. Similar to cortisol, catecholamines increase at birth also promoting successful fetal adaptation of blood pressure, energy metabolism, and thermogenesis^3^. Oxytocin, a natural and iatrogenically administered hormone, surges promoting uterine contractions in labor. After birth, oxytocin stimulates lactation and supports maternal-infant bonding through integration with limbic pathways^4^. These and other substances are potentially neuroactive, yet the effects of mode of delivery and exposure to labor on autonomic tone and cortical activity in the term newborn remains incompletely understood.

Improving understanding of normal early post-transition ANS tone and electrocortical function may provide a useful reference for studies of high-risk infant groups and may have prognostic value. For example, preterm newborns, an important high-risk newborn group with a potential for significant neurodevelopmental and psychological long-term impairment, have reduced autonomic tone compared to term newborns^5^. Therefore, it is important to understand how labor and mode of delivery influence measures of electrocortical and brainstem ANS function in low-risk term newborns, who otherwise would not be expected to have physiological alterations in these systems.

Brainstem ANS function can be measured non-invasively by analysis of heart rate variability (HRV) while electrocortical function can be measured by an electroencephalogram (EEG). The primary objective of this study was to determine whether there is a difference in HRV metrics and EEG power in low-risk term newborns according to the route of delivery and exposure to labor. We hypothesized that infants with exposure to labor, either during vaginal delivery or caesarean section following a period of labor, would have higher autonomic tone and higher EEG power compared to infants delivered by elective caesarean section prior to the onset of labor.

## Methods

We performed a prospective cohort study at Inova Women's Hospital, Fairfax, VA of low-risk term (≥37 weeks gestational age) newborns from uncomplicated singleton pregnancies and deliveries between May 2017 to June 2018. Newborns were enrolled and studied at <72 hours of age and prior to hospital discharge in the mother-baby unit after obtaining informed consent. Newborns were considered to be low-risk based on maternal, fetal, and neonatal parameters and were excluded if there was evidence of perinatal depression with 1-minute Apgar less than 7 and/or need for resuscitation at delivery, significant maternal illness or complications during pregnancy (i.e. diabetes, hypertension, substance abuse, etc.), small or large for gestational age weight (birth weight <10th percentile or >90th percentile), known brain malformation, dysmorphic features or congenital anomalies suggestive of a genetic or a metabolic syndrome. Newborns were also excluded if their clinical caretakers recommended observation in the neonatal intensive care unit (NICU). Thus, enrolled newborns were cared for in a regular newborn nursery and/or at the mother’s bedside. Following analysis of HRV and EEG, newborns were excluded if their data was of insufficient quality or duration. The study was approved by the Institutional Review Board at Inova Fairfax Hospital, Fairfax, VA and Children’s National Health System, Washington, DC and was performed in accordance with relevant guidelines and regulations. Study data were collected and managed using REDCap electronic data capture tools hosted at Children’s National Medical Center, Washington, DC, USA^6^.

Demographic and clinical characteristics were recorded for each mother-infant pair. The mode of delivery was categorized into three groups as vaginal delivery (VD), cesarean section (CS) after labor (L-CS), or as elective CS (E-CS) without preceding labor. Vaginal deliveries occurred either with or without augmentation (pitocin [oxytocin] and/or artificial rupture of membranes) and with or without epidural or spinal analgesia. For infants delivered by L-CS or E-CS, the indication for CS was documented. None of the CS deliveries were performed with general anesthesia. Data on the duration of labor and the duration of rupture of membranes was not available.

### ECG and HD-EEG recording

Recording sessions were scheduled between feedings and caregiving events, at a time when the infant would be more likely to sleep. The infant was swaddled and placed in a bassinette. For each infant, simultaneous recordings of high-density (HD) EEG and electrocardiogram (ECG) were acquired using the 128-electrode HD Hydrocel Geodesic Sensor Net (Electrical Geodesics Inc., Eugene, OR, USA) for up to 60 minutes. Standard electrode net application procedures were followed for electrode net preparation and application and impedances were checked prior to the start of the recording. During the study, the HD-EEG setup was updated and this changed the sampling rate from 1000 Hz to 250 Hz. Data measured at 1000 Hz were then downsampled to 250 Hz for analysis so all analyses were done using 250 Hz. Prior to 10/15/17, EEG was recorded using Net Station5.4, and after 11/17/17, it was recorded using Net Station5.4.1.1 software (Electrical Geodesics Inc. [EGI], Eugene, OR) on a MacBook Pro laptop computer. ECG was simultaneously acquired using Physiological Input Box (EGI) and Physio 16 (EGI) devices.

### ECG processing and HRV data analysis

HRV and HD-EEG data were analyzed at Children’s National Health System, Washington, DC in the Advanced Physiological Signal Processing Laboratory. ECG was bandpass filtered between 0.5–70 Hz to attenuate the baseline drift and the R-wave (the wave with the dominant amplitude in each cardiac cycle) was identified^7^. Beat-to-beat interval (RRi) was calculated. Artifacts such as missed and/or extra beats were removed^8^. A one second ECG signal was considered artifact-free if the standard deviation is greater than 0.01 millivolts. The RRi were partitioned into non-overlapping 10-min epochs. For spectral analysis, the RRi were converted into evenly sampled data using a cubic spline interpolation technique with a sampling rate of 5 Hz.

For each subject, heart rate (HR) was calculated and HRV was analyzed in the time domain (alpha1, alpha2, root mean square 1 [RMS1] sec, and root mean square 2 [RMS2] sec) and by frequency domain metrics (normalized low frequency [nLF], normalized high frequency [nHF], low frequency [LF] dB, and high frequency [HF] dB) in 10-min epochs and averaged over the composite of all epochs^7^. The following metrics characterize the sympathetic tone: alpha1, RMS1 (sec), RMS2 (sec)^9–11^, and HF (dB) and nHF characterize the parasympathetic tone^9^. LF (dB) and nLF power reflect both sympathetic and parasympathetic mediated activity. Alpha2 characterizes ultraslow changes in the heart rate, and is below the frequency of sympathetic tone.

### Detrended fluctuation analysis – time domain characterization

Detrended fluctuation analysis is a modified RMS analysis approach and has been described previously^5,12^. Using the global fluctuation function we calculated RMS1 (sec) as the maximum value of the global fluctuation function for ‘s’ between 15–50 beats and RMS2 (sec) as the maximum of the global fluctuation function for ‘s’ between 100–150 beats^13^. We also calculated the *α* exponent from the slope of the global fluctuation function versus ‘s’ in double logarithmic representation. *α*_1_ was obtained from the region 15–30 beats (short term scale) and *α*_2_ was obtained from the region 35–150 beats (long term scale/ultralow frequency)^13^. The RMS (sec) characterizes the variability in the RRi whereas the *α* metrics characterize the autocorrelation in the RRi.

### Spectral analysis

We used the Welch periodogram approach to estimate the power spectrum^5^. For RRi in each 30 sec epoch, the periodogram was calculated as the square of the magnitude of the Fourier transform of the data and we estimated the power spectrum as the average of the periodograms over all epochs. We then determined the spectral powers in LF (dB) and HF (dB) as the median of the logarithm of the power in 0.05–0.25 Hz and 0.3–1 Hz frequency bands, respectively. nLF power was calculated as the ratio of the sum of the powers in the 0.05–0.25 Hz band to the total power and nHF power as the ratio of the powers in 0.3–1 Hz band to the total power. We defined total power as the sum of the powers from 0.05–2 Hz.

### Asymmetry Index

To characterize the accelerations and decelerations in the HR, we calculated the asymmetry index^14^, following the method by Kovatchev *et al*.^14^, by using RRi as follows: we identified RRi greater than the median value of RRi and denoted them as PRRi (positive part of the distribution)^14^. We calculated an index R1 as the mean of the square of the deviation of PRRi from the median RRi. Similarly, we calculate RRi values less than the median value of RRi and denoted them as NRRi (negative part of the distribution). We calculated an index R2, as the mean of the square of the deviation of NRRi from the median RRi. To this end, the asymmetry index was calculated as the ratio of R1 to R2.

### HD-EEG Data analysis

The EEG data were exported to MATLAB format offline using Net Station 5.4 or 5.4.1.1 software. The EEG data were first high pass filtered using a 0.3 Hz Butterworth filter of order 4, and then notch filtered at 60(58–62) Hz. The EEGs contaminated with ECG were cleaned using a frequency-based approach^15^. A one second EEG was considered artifact-free if the standard deviation is between 0.01 and 50 microvolts (mcV) and its absolute amplitude is less than 250 mcV. Electrodes with artifact >30 percent of the time were removed from analysis. Newborn’s state was classified into quiet and active based on HR acceleration^16,17^. We considered newborns who had at least one 5 minute EEG in both active and quiet state to be of high-quality. The Welch periodogram approach was used to estimate the power spectrum in 5-minute segments. For each 3-second epoch of the EEG, the periodogram was calculated as the square of the magnitude of the Fourier transform of the data. The power spectrum was estimated as the average of the periodograms over all epochs. EEG absolute power was measured in delta (0.5–4 Hz), theta (4–8 Hz), alpha (8–13 Hz), beta (13–30 Hz), and gamma (80–100 Hz) frequency bands in both quiet and active states. The median absolute power of artifact free electrodes was considered for further analysis. For newborns that had more than one 5-minute segment, the median of all segments was considered for analysis. Similarly, relative power was calculated by dividing the absolute power value with the total power (0.5 to 110 Hz] for quiet and active states. For every 5-minute EEG segment in quiet and active states, the global connectivity index was calculated for delta, theta, alpha, beta, and gamma frequency bands^15^. The connectivity indexes were averaged in the quiet/active states.

### Statistical analysis

Kruskal-Wallis tests were done for continuous clinical variables. Multiple quantile regression analyses were conducted using qreg in stata 15.1 (StataCorp LLC, College Station, TX, USA) to evaluate the difference in predicted median levels of HRV and EEG parameters by mode of delivery^18–20^. The models were controlled for gestational age at birth, hour of age at recording, and percent active status.

## Results

Of a total of 167 term newborn enrollees, 118 (71%) provided either HRV or HD-EEG assessments considered of high quality for analysis and study inclusion. The demographic and clinical characteristics of the 118 newborns are shown in Table 1. The infants were born at a median of 39.3 (IQR 39.0–40.0) weeks gestational age and were studied at 23.1 (IQR 17.5–28.9) hours of age. Sixty-two (52.5%) infants were born by VD, 22 (18.6%) by L-CS, and 34 (28.8%) by E-CS. The infant groups were similar for weight, gender, and one- and five-minute Apgar scores. Newborns born by L-CS and E-CS were studied at an older postnatal age in hours than those born by VD (P = 0.0001). Twenty (90.9%) infants born by L-CS had CS for failure to progress or for failed induction of labor and two (9.1%) had another reason (fetal intolerance to labor or presence of meconium). Of 34 infants delivered by E-CS, 29 (85.3%) were due to prior maternal CS delivery, 3 (8.8%) were due to breech presentation, and 2 (5.9%) were other reasons (placenta previa or cerclage).

**Table 1:** Clinical and Demographic Characteristics of low–risk term cohort by mode of delivery.

Characteristic Median (IQR) or N (%)	Overall (n = 118)	VD (n = 62)	L–CS (n = 22)	E–CS (n = 34)	p-value
Maternal age	32 (30–34)	31.5 (30–35)	30 (27–32)	33.5 (32–35)	0.0003
Maternal BMI	28.7 (31.3–26.5)	28.0 (26.0–30.5)	30.8 (26.6–33.9)	30.1 (28.1–31.3)	0.04
**Race**					
American Indian/Alaskan Native	1 (0.9)	0 (0)	1 (5.3)	0 (0)	0.07
White	74 (70.5)	42 (73.7)	10 (52.6)	22 (75.9)	
Asian	20 (19.1)	12 (21.1)	3 (15.8)	5 (17.2)	
Black or African American	7 (6.7)	3 (5.3)	3 (15.8)	1 (3.5)	
Native Hawaiian or Other Pacific Islander	0 (0)	0 (0)	0 (0)	0 (0)	
Other (specify)	3 (2.9)	0 (0)	2 (10.5)	1 (3.5)	
**Ethnicity**					
Hispanic or Latino	15 (12.7)	6 (9.7)	4 (18.2)	5 (14.7)	0.50
Para ≥1 (Y/N)	111 (94.9)	58 (93.6)	19 (90.5)	34 (100)	0.21
Birth GA (weeks)	39.3 (39–40)	39.5 (39.1–40.1)	39.9 (39.1–40.4)	39.1 (39.0–39.3)	0.0005
Birth weight (grams)	3345 (3120–3590)	3397.5 (3180–3650)	3360 (3110–3575)	3195 (3100–3590)	0.40
Birth HC	34.9 (33.7–35.6)	34.85 (33.7–35.6)	34.3 (33–35.6)	34.9 (34.3–35.6)	0.39
Birth length	52 (50.8–53.3)	52.05 (50.8–53.3)	52.04 (50.8–53.3)	51.4 (50.2–53.3)	0.56
Gender male	57 (48.3)	26 (41.9)	15 (68.2)	16 (47.1)	0.11
Apgar score 1 min	8 (8–9)	8 (8–8)	8 (8–9)	8 (8–9)	0.07
Apgar score 5 min	9 (9–9)	9 (9–9)	9 (9–9)	9 (9–9)	0.79
Age at recording (hours)	23.1 (17.5–28.9)	18.8 (16.5–24.7)	29.8 (13.7–43.8)	27.1 (22.7–30.2)	0.0001
Duration of recording (minutes)	48 (42–52)	47.5 (42–52)	50 (45–56)	48 (41–50)	0.28
Usable HRV data	95 (80.5)	51 (82.3)	16 (72.7)	28 (82.4)	0.59
Usable HD–EEG data	101 (85.6)	50 (80.7)	21 (95.5)	30 (88.2)	0.21

Abbreviations: VD – vaginal delivery, L–CS– caesarean section in labor, E–CS– elective caesarean section, BMI – body mass index, GA – gestational age, HC – head circumference, HRV – heart rate variability, HD–EEG– high–density electroencephalogram.

By time and frequency domain HRV analysis, there were no differences in HRV metrics between the three modes of delivery (Table 2). The asymmetry index was higher in infants delivered by L-CS compared to VD and E-CS (P = 0.03, Table 2). We found no difference in EEG spectral powers by route of delivery, except that newborns delivered by L-CS had lower relative gamma power compared to those born by VD and E-CS (P = 0.005, Table 3).

**Table 2:** HRV metrics by mode of delivery by a factor of 100.

HRV metric	Overall (n = 95)	VD (n = 51)	L–CS (n = 16)	E–CS (n = 28)	p-value
Alpha 1	139.1 (129.6–148.6)	131.4 (118.9–143.9)	153.8 (129.2–178.3)	144.8 (125.5–164.0)	0.20
Alpha 2	120.2 (115.5–124.9)	121.3 (114.5–128.1)	116.8 (99.0–134.5)	120.3 (114.5–126.1)	0.89
RMS 1 (sec)	2.8 (2.5–3.1)	2.8 (2.3–3.3)	2.9 (2.2–3.5)	2.8 (2.3–3.3)	0.99
RMS 2 (sec)	10.7 (9.8–11.6)	11.1 (9.8–12.3)	10.4 (8.2–12.6)	10.3 (8.8–11.9)	0.71
LF (dB)	−275.7 (−284.7 – −266.7)	−275.6 (−288.8 – −262.4)	−262.8 (−277.7 – −247.8)	−283.1 (−300.1 – −266.2)	0.15
HF (dB)	−374.6 (−384.8 – −364.4)	−376.9 (−391.2 – −362.6)	−363.7 (−390.2 – −337.2)	−376.7 (−395.6 – −357.7)	0.67
nLF	72.5 (70.0–75.0)	72.2 (68.9–75.5)	73.2 (67.4–79.0)	72.5 (68.0–77.1)	0.95
nHF	22.5 (20.1–25.0)	23.5 (20.2–26.8)	22.9 (16.7–29.1)	20.7 (16.8–24.5)	0.56
Asymmetry index	121.8 (113.1–130.5)	117.0 (105.1–128.9)	155.5 (126.3–184.6)	111.4 (97.5–125.2)	0.03

^*^Median (95% Confidence Interval).

**Table 3:** EEG Power and connectivity index (CI) multiplied by a factor of 1000 by mode of delivery.

EEG Measure	Overall (n = 101)	VD (n = 50)	L–CS (n = 21)	E–CS (n = 30)	p-value
**Absolute power**					
Delta Q_A	−7.4 (−26.5–11.6)	−7.5 (−37.6–22.5)	5.1 (−24.6–34.8)	−16.0 (−51.9–20.0)	0.63
Theta Q_A	−10.4 (−28.0–7.1)	−14.7 (−41.3–11.8)	7.2 (−31.5–45.9)	−15.7 (−48.8–17.5)	0.58
Alpha Q_A	−21.6 (−38.0 – −5.2)	−21.3 (−48.7–6.0)	−37.3 (−70.9 – −3.6)	−11.1 (−40.7–18.4)	0.51
Beta Q_A	−51.1 (−68.0 – −34.2)	−55.5 (−72.5 – −38.5)	−64.1 (−103.2 – −24.9)	−34.7 (−77.9–8.5)	0.57
Gamma Q_A	−133.6 (−139.8 – −87.4)	−119.0 (−149.8 – −88.2)	−152.7 (−195.3 – −110.0)	−77.2 (−142.8 – −11.7)	0.11
**Relative power**					
Delta Q_A	22.5 (14.9–30.2)	24.4 (12.5–36.3)	19.2 (4.0–34.4)	21.8 (6.4–37.2)	0.86
Theta Q_A	2.8 (1.6–3.9)	3.2 (1.8–4.6)	2.3 (−0.6–5.2)	2.3 (0.0–4.6)	0.76
Alpha Q_A	0.3 (−0.1–0.7)	0.5 (−0.1–1.2)	−0.1 (−0.9–0.8)	0.2 (−0.7–1.1)	0.54
Beta Q_A	−0.2 (−0.6–0.3)	−0.2 (−0.9–0.5)	−0.3 (−1.2–0.5)	0.0 (−0.6–0.7)	0.76
Gamma Q_A	−0.5 (−0.7 – −0.3)	−0.6 (−0.9 – −0.3)	−0.9 (−1.1 – −0.7)	−0.2 (−0.6–0.2)	0.005
**Quiet CI**					
Delta CI	475.3 (442.8–507.8)	461.3 (420.1–502.4)	471.2 (398.1–544.2)	501.5 (432.0–570.9)	0.64
Theta CI	405.1 (374.5–435.7)	403.5 (357.6–449.4)	426.1 (365.0–487.3)	392.9 (326.8–459.0)	0.74
Alpha CI	354.7 (324.4–385.1)	350.6 (310.3–390.9)	364.5 (278.4–450.6)	354.9 (291.6–418.2)	0.96
Beta CI	308.1 (280.9–335.2)	314.9 (277.7–352.0)	285.4 (229.0–341.7)	312.7 (252.6–372.8)	0.69
Gamma CI	148.7 (137.4–160.1)	155.1 (140.3–167.8)	139.1 (124.2–154.0)	145.0 (117.4–172.5)	0.32
**Active CI**					
Delta CI	426.1 (386.7–465.5)	412.5 (369.7–455.2)	401.0 (302.5–499.4)	466.3 (377.8–554.7)	0.51
Theta CI	364.9 (328.6–401.2)	368.5 (318.6–418.5)	352.6 (275.5–429.7)	367.4 (287.7–447.1)	0.94
Alpha CI	335.2 (300.8–369.6)	355.0 (305.0–405.0)	307.3 (237.5–377.1)	321.7 (242.9–400.5)	0.55
Beta CI	283.4 (258.7–308.0)	305.0 (269.4–340.7)	257.5 (223.6–291.4)	265.3 (209.5–321.1)	0.20
Gamma CI	174.1 (161.4–186.9)	177.0 (153.9–200.1)	167.5 (147.5–187.5)	174.0 (152.8–195.1)	0.80

Q = quiet; A = active; CI = connectivity index.

*Median (95% Confidence Interval).

## Discussion

In this prospective cohort of low-risk term newborn infants, we found that the mode of delivery and exposure to labor or no labor did not significantly alter early ANS tone measured by time and frequency domain metrics. However, using a previously described measure of asymmetry in the heart rate, the asymmetry index^14^ showed that infants born by caesarean section following a period of labor (L-CS) had significantly increased accelerations in their heart rate (a higher asymmetry index). Also, in the group of infants born by caesarean section during labor, we found significantly lower relative gamma frequency EEG activity compared to infants born by vaginal delivery or elective caesarean section (P = 0.005). Together, these findings may indicate an increased stress response and arousal difference in newborns with this mode of delivery compared to either vaginal delivery or elective caesarean section, without a difference in brainstem ANS function. Given the paucity of data on the effects of mode of delivery on HRV metrics in low-risk term newborns, the results help to fill an important knowledge gap in this field and provide a normative reference for studies in high-risk infant groups.

In our study, at a median of 23.1 hours of age, healthy term newborns did not demonstrate a difference in either sympathetic or parasympathetic tone by mode of delivery. This finding differs from that in a recent study by Kozar *et al*.^21^, comparing infants born by caesarean section under general anesthesia with babies born vaginally without anesthesia. In these infants, studied within the first few hours after delivery, the authors found significantly higher LF variability and lower HF variability in those delivered by cesarean section compared to infants born vaginally. These differences resolved by the third to fourth postnatal day^21^. The infants in our study differed in that all caesarean section deliveries were performed without general anesthesia and most vaginal deliveries were accompanied by epidural or spinal analgesia. It is not well established how maternal general anesthesia affects ANS tone in the newborn. In a study comparing maternal and neonatal outcome of general anesthesia versus epidural anesthesia for caesarean section, no difference was seen in Apgar scores or neurological adaptive capacity scores at two and 24 hours after delivery^22^. In a different study to evaluate ANS function in the term newborn following non-reassuring fetal heart rate tones in labor, Sheen and colleagues compared newborns born via vaginal delivery versus caesarean section^23^. They found that newborns born by caesarean section had lower ANS tone compared to those born by vaginal delivery, especially in the infants with caesarean section following non-reassuring fetal heart tones^23^. The reason for this disparity between the Sheen study and ours may be a difference in clinical indication of caesarean section; only 1 of our 56 (1.8%) infants born via caesarean section had non-reassuring fetal heart tones reported prior to cesarean delivery compared to 22 of 83 (26.5%) in their study^23^. Non-reassuring fetal heart tones are associated with fetal hypoxemia and may cause a decrease in HRV due to vagal response^24^. Infants without exposure to fetal hypoxemia prior to caesarean section would thus be less likely to have attenuation in autonomic tone prior to delivery.

Postnatal age at the time of testing may also contribute to differences in early autonomic tone. Systolic blood pressure and SpO_2_ increase by the third and fourth postnatal day in infants delivered both by vaginal delivery and by caesarean section, indicating a postnatal transitional period of maturation of the ANS, cardiovascular, and respiratory systems that extends for a few days beyond delivery^21^. Similarly, when ANS tone is evaluated both within a few hours of birth and at three to four days of age, there is a maturational increase in HRV metrics^21^. In a low-risk otherwise healthy term newborn, any minor difference in ANS tone due to general anesthesia, mild fetal distress, or other inconsequential exposures around the time of delivery seems to dissipate within a few days of birth and does not seem to have a sustained influence on ANS tone^21^. Thus, it is possible that there may be immediate, but limited effects on infant HRV according to both the circumstances of birth and the timing of testing.

Heart rate is controlled in balance by regulation of vagal stimulation and vagal withdrawal to decrease and increase the heart rate, respectively, along with mechanical reflexes and stimulation or inhibition of sympathetic activity^25^. The asymmetry index provides a measure of the contributions of heart rate accelerations and of decelerations to the heart rate pattern^14^. Asymmetry analysis of heart rate patterns may be a marker of early sepsis in the newborn^14^. Using this metric, infants with sepsis showed higher sample asymmetry (i.e. increased accelerations) one day before sepsis^14^. Similarly, infants born via caesarean section following labor in our cohort, had a higher asymmetry index (i.e. increase in heart rate accelerations) compared to the other mode of delivery groups. This finding may indicate an acute stress response and higher sympathetic activity that was not detected by a difference in HRV time and frequency domain analysis, since none of our infants had sepsis or other clinical complications at delivery. The reason for the difference in the L-CS group may be due to the combined stress of labor and abdominal delivery compared to a single stress of labor and vaginal delivery or of abdominal delivery without labor in the other two groups.

We measured EEG power using the HD-EEG system in a range of frequencies and found a difference by mode of delivery in only the gamma range. In our study, infants delivered by caesarean section following labor had lower relative gamma power compared to both vaginal delivery and elective caesarean section groups. Lower gamma activity in this infant group with concomitant increase in asymmetry index (in this same group) may be due to acute stress induced impairment in the network involved in the arousal of the pedunculopontine nucleus in the brainstem^26^. Gamma band activity is known to increase with wakefulness and learning^27^. In our study, we focused on a higher gamma range of 80–100 Hz^28^. One reason was to avoid broad range of line noise, which can be an issue in the hospital environment and may cause contamination in the quantification of gamma power. While gamma activity has not specifically been evaluated by mode of delivery in prior studies to our knowledge, EEG sleep states have been investigated within the first few hours of age and show a difference in duration of active and quiet sleep states between vaginal delivery, elective caesarean section, and emergent caesarean section deliveries^29^, but no significant difference in the wakeful state^29^.

Oxytocin, vasopressin, and neurosteroids have important roles in the preparation of the fetal brain and the cardiovascular and respiratory systems for labor and postnatal transition to the extrauterine environment^4,30^. The route of delivery, exposure to labor, gestation at delivery and neonatal birth asphyxia may affect levels of the stress hormones, neurosteroids, vasopressin and cortisol^4,30^. This is a complex issue to consider regarding the mode of delivery and exposure to labor; however our findings do not suggest a major effect of labor versus no labor on electrocortical and brainstem autonomic function in low-risk newborns at term.

This study is limited by a difference in timing of recordings between infant groups. Healthy term newborns born via caesarean section typically remain in the birth hospital for around 48 hours, while many vaginal deliveries are discharged within 24 hours of birth providing us a longer time to enroll and study newborns following caesarean section. Accurate record of duration of labor and fetal heart rate pattern was not available. The study was also not able to evaluate the role of sleep-wake cycles in HRV for any difference in the mode of delivery due to the limited time between our study and newborn clinical care, e.g. feedings. Infant irritability and lack of consistent sleep and wakeful states affected signal quality in some infants, resulting in some cases having data of insufficient quality. However, compared to other studies comparing modes of delivery, our study was benefitted by access to a large volume delivery hospital with a high volume of elective caesarean sections.

## Conclusion

In a low-risk term newborn cohort, the ANS and cortical systems seem to function well during transition regardless of mode of delivery and exposure to labor. Infants delivered by caesarean section following a period of labor however, may have an acute stress response which is evident by a difference in asymmetry index and relative EEG gamma power, despite a lack of difference in measures of autonomic tone or other EEG frequencies at one day of age. Evaluation of these differences at a later time point is needed.
